# How previous experience shapes future affective subjective ratings: A follow-up study investigating implicit learning and cue ambiguity

**DOI:** 10.1371/journal.pone.0297954

**Published:** 2024-02-09

**Authors:** Fiorella Del Popolo Cristaldi, Giulia Buodo, Filippo Gambarota, Suzanne Oosterwijk, Giovanni Mento

**Affiliations:** 1 Department of General Psychology, University of Padua, Padua, Italy; 2 Department of Developmental Psychology and Socialization, University of Padua, Padua, Italy; 3 Department of Social Psychology, University of Amsterdam, Amsterdam, The Netherlands; 4 Amsterdam Brain and Cognition Centre (ABC), Amsterdam, The Netherlands; 5 Scientific Institute, IRCCS E. Medea, Conegliano, Treviso, Italy; Wilfrid Laurier University, CANADA

## Abstract

People use their previous experience to predict future affective events. Since we live in ever-changing environments, affective predictions must generalize from past contexts (from which they may be implicitly learned) to new, potentially ambiguous contexts. This study investigated how past (un)certain relationships influence subjective experience following new ambiguous cues, and whether past relationships can be learned implicitly. Two S1-S2 paradigms were employed as learning and test phases in two experiments. S1s were colored circles, S2s negative or neutral affective pictures. Participants (Experiment 1 N = 121, Experiment 2 N = 116) were assigned to the certain (CG) or uncertain group (UG), and they were presented with 100% (CG) or 50% (UG) S1-S2 congruency during an uninstructed (Experiment 1) or implicit (Experiment 2) learning phase. During the test phase both groups were presented with a new 75% S1-S2 paradigm, and ambiguous (Experiment 1) or unambiguous (Experiment 2) S1s. Participants were asked to rate the expected valence of upcoming S2s (expectancy ratings), or their experienced valence and arousal (valence and arousal ratings). In Experiment 1 ambiguous cues elicited less negative expectancy ratings, and less unpleasant valence ratings, independently of prior experience. In Experiment 2, both groups showed similar expectancies, predicting upcoming pictures’ valence according to the 75% contingencies of the test phase. Overall, we found that in the presence of ambiguous cues subjective affective experience is dampened, and that implicit previous experience does not emerge at the subjective level by significantly shaping reported affective experience.

## Introduction

### Emotions as predictions

People use previous experience to predict future affective events. For example, imagine that your neighbor has a medium-sized dog with brown spotted fur that always snarls at you, causing you fear and annoyment. Imagine now that you are having lunch with your best friend, who is introducing you to their recently adopted little dog. As the dog enters the room running briskly, you seem to see that his fur is brownish, and perhaps spotted. Does your past annoying experience with your neighbor’s dog make you think that your best friend’s new dog is extremely unfriendly, just because the two dogs look alike? When situations like this happen in real life, predictions play a crucial role. Based on your past experiences your brain generalizes the concept of unfriendliness, developed on your neighbor’s dog, also to new, perceptually similar dogs, predisposing you to feel fear and annoyment. All these computations occur quickly and without conscious awareness. In other words, affective predictions (and the associated subjective experience) must generalize from the specific features of past contexts (from which they are implicitly learned) to new and potentially ambiguous contexts (i.e., contexts that look alike past ones, but have different perceptual features), to be effective in promoting survival and allostatic balance with the environment [1,2]. However, the specific mechanisms by which this process occurs remain unclear.

According to predictive models of emotion [3,4], affective predictions are constructed along three distinct neurocomputational stages: prediction *generation*, in which prior experience is combined with present information to construct affective predictions; prediction *implementation*, in which predictions are used to pre-arrange the best action plans to deal with the expected situation; and prediction *updating*, in which current environmental inputs are compared with predictions, and in case of mismatch the unexpected information (encoded as a *prediction error*) acts as a feedback to adjust subsequent predictions. Predictive models are assumed to be (i) *probabilistic*, since they encode the statistical regularities within the observed inputs [1,2,5]; (ii) *generative*, because they generalize across sensory modalities, contexts, and time [1,2]; and (iii) *implicit*, since they act mostly outside of awareness, potentially emerging into it only in case of a prediction violation [6]. It follows that human brains do not merely react to affective stimuli at the time of their occurrence, but rather, as a spontaneous activity, they are constantly busy in generating-implementing-updating predictions in the service of allostasis [7,8].

Prediction construction is assumed to represent the core mechanism on which the brain relies, in order to provide the body with optimal resources for growth, adaptation to the environment, and survival [1,5,7–9]. Previous experience (i.e., knowledge derived from the extraction of statistical regularities of stimuli occurrence in the past) plays a pivotal role in the construction of new affective predictions: it interacts with momentary information (e.g., physical properties of the environment, contingencies experienced between present stimuli) in constraining and refining the pool of information used to generate predictions [1,3,5,6]. However, predictive processing’s assumptions about (i) whether contingency learning (i.e., extracting statistical regularities of stimuli co-occurrence) may develop implicitly and (ii) whether these learnings generalize to new, and potentially ambiguous contexts are to date mainly based on empirical evidence related to cognitive domains such as visual perception [10–12] or motor control [13,14]. Little experimental evidence about the application of these assumptions to the affective domain has been collected so far.

To better understand how affective predictions are related to subjectively experienced affective states, predictive models of emotion [3,4] have reconceptualized subjective affective experience within their framework. Subjectively experienced affective states have been defined as the representation of valence (i.e., pleasantness/unpleasantness), arousal (i.e., activation/calm) or even discrete emotions (e.g., anger, fear) in subjective awareness, on which an individual can verbally self-report [15]. Predictive models of emotion [3,4] assume that affective experience derives from the brain’s spontaneous predictive activity, and have redefined it as the active process of making meaning of present stimuli by predicting and categorizing them on the basis of past experiences [3]. When this process draws on conceptual emotion knowledge, the resulting predictive models and their associated affective responses can be subjectively experienced as emotions [3,16,17]. It follows that subjective affective experience may be sensitive to statistical regularities experienced in the past. For example, the more reliable the regularities, the more expectancies about the valence of upcoming stimuli will draw on them to predict upcoming stimuli [18]. However, it is still unknown whether previously learned probabilities may influence subjective affective experience when facing new, potentially ambiguous cues (i.e., ambiguous signals preceding the occurrence of an affective stimulus). Also, it is not clear if affective environmental contingencies might be inferred from past experience at an implicit level (i.e., without explicit awareness, and without focusing attention on contingencies themselves at the time of their occurrence), and if this may ultimately shape subjective affective experience during future predictions. The present research aimed to test whether these two crucial assumptions of predictive processing models apply to the affective domain by employing a methodological replication approach. We draw on our previous work [18] to investigate (i) if being exposed to past (un)certain contingencies might influence future subjective affective ratings to new and potentially ambiguous cues (i.e., cues with different perceptual properties from the ones previously experienced); and (ii) if people are able to implicitly extract (un)certain probabilistic information, and use it later to subjectively predict new affective events.

### A novel experimental paradigm

To study the effects of previous experience on affective predictions we recently developed a novel experimental paradigm [18], which integrates the logic of traditional emotional S1-S2 paradigms (see [19] for a review) with an uninstructed learning component. In this novel paradigm two separate emotional S1-S2 paradigms are employed as a *learning* and *test* phase, respectively. As in typical emotional S1-S2 paradigms, a sequential presentation of two stimuli is implemented (both in the learning and test phases): the S1 (or *cue*) is a symbolic stimulus (manipulated at two levels, i.e., red or blue circles) preceding the occurrence of an affective stimulus, while the S2 (or *target*) is an emotional stimulus (manipulated at two levels, i.e., negative or neutral affective pictures). The sequence of events in the S1-S2 paradigm allows to target the three stages of affective prediction construction: the S1 reflects the *generation* stage, the inter-stimulus interval (ISI) between S1 and S2 the *implementation* stage, and the S2 the *updating* stage [20]. The contribution of our new paradigm is that it manipulates *actual previous experience* through *uninstructed* certain vs. uncertain probabilistic contingencies between S1 and S2, experienced during a separate learning phase. This approach is markedly different from extant S1-S2 paradigms that manipulate *explicitly labeled* probabilistic information while participants perform the task.

During the learning phase of this paradigm, previous experience is manipulated between subjects by dividing participants into two experimental groups: the *certain* group (CG) and the *uncertain* group (UG). According to the group, they are presented with a 100% (CG) or 50% (UG) S1-S2 congruency, namely the probabilistic ratio between S1 color and S2 valence they are exposed to. During the test phase, all participants are then presented with a new S1-S2 paradigm with a fixed 75% S1-S2 congruency (see [18] and below for a detailed explanation of the choice to use the 75% S1-S2 ratio in the learning phase). In the test phase, they are asked to rate their subjective affective experience in terms of either the expected valence of upcoming S2s (*expectancy ratings*), or the experienced valence and arousal to S2s (*valence* and *arousal ratings*). Participants are left uninstructed about the probabilistic ratios they are exposed to during the whole paradigm.

### Inconsistent evidence on subjective affective experience

Extant literature has collected inconsistent evidence with regard to the effect of (un)certain stimulus predictability on subjective affective ratings. As for expectancy measures related to the *generation*-*implementation* stages, our previous study [18] showed that experiencing certain contingencies (100%) during the learning phase subsequently elicited more extreme expectancy ratings (i.e., participants predicted the valence of future stimuli according to previously learned contingencies), and this effect generalized from the visual to the auditory sensory modality. Other studies implementing typical S1-S2 paradigms found negatively-biased expectancies (i.e., an overestimation of negative S2s occurrence) in the uncertain (50%) condition [21–25]; and that ambiguous cues (i.e., cues with uninstructed 50% predictive value) elicited less negative expectancy ratings than unambiguous cues [26].

Regarding valence and arousal ratings measured during the *updating* stage, we did not find any effect of previous experience [18], consistently with some studies implementing typical S1-S2 paradigms that found no effect of current (un)certainty on valence ratings [21–24,27,28]. Other traditional S1-S2 studies, instead, showed a more intense subjective experience either in the certain (100%) [27,29–32] or in the uncertain (50%) condition [33], and also that ambiguous cues elicited more unpleasant mood ratings [26]. Another study [23] compared an explicit anticipation condition (in which participants were asked for expectancy ratings) with an implicit anticipation condition (in which participants were asked for a target detection task): no effects of cue predictive meaning (100% vs. 50%) emerged on accuracy to the target detection task, faster reaction times (RTs) were found in the certain condition, and no significant results were found on S2-valence ratings.

### A follow-up research

Overall, evidence on the effects of past knowledge on subjective affective experience remains fragmentary, with our previous work [18] suggesting that a reliable (i.e., certain) previous experience affects future expectancies, and S1-S2 studies suggesting that either certain [27,29–32] or uncertain [21–26,30,33] contingencies can lead to an intensification of the related affective experience. Besides, little is known yet about the potential effects of cue ambiguity on new affective predictions, nor on the actual possibility to infer affective environmental contingencies implicitly. Both these factors are nonetheless crucial to construct efficient affective predictions. In fact, since our environments are characterized by frequent changes, affective predictions must be flexible in adapting to new, and potentially ambiguous, contextual features [3,6,8]. Moreover, in daily life people should be able to spontaneously learn contingencies from the environment, and to use them as priors for subsequent affective predictions, even in absence of explicit instructions and/or awareness of the contingencies themselves [6]. However, despite the importance of both of these aspects for constructing efficient predictions, it still lacks solid experimental evidence on how they may influence subjective affective experience.

Based on this, in the present research we implemented two follow-up experiments (pre-registered on the Open Science Framework—OSF; Experiment 1: https://osf.io/gdr3b/, Experiment 2: https://osf.io/z5esb/), in which we modified some features of our former paradigm [18]. In particular, in order to investigate the construction of new affective predictions as a function of cue ambiguity, in Experiment 1 we introduced ambiguous cues in the test phase. Here, unbeknownst to participants, we presented two new reddish and bluish S1 colors (i.e., coral and turquoise, here defined as ambiguous) in addition to those already presented during the learning phase (i.e., red and blue, here defined as unambiguous). Further, to test if the probabilistic information available in the environment can be extracted and learned at an implicit level (i.e., not explicitly focusing attention on the probabilistic relationships between stimuli), in Experiment 2 we engaged participants in a distracting task (i.e., a parity judgment task) during the learning phase.

According to predictive models of emotion [3,4], we hypothesized that participants would generalize previously learned contingencies to new ambiguous cues in Experiment 1, and that they would infer affective environmental contingencies without explicitly focusing attention (and use them later to predict future stimuli) in Experiment 2.

## Experiment 1

### Method

Experiment 1 investigated whether (un)certain past experience might influence future subjective affective ratings as a function of cue ambiguity. We pre-registered the study on Open Science Framework (OSF) (https://osf.io/gdr3b/).

According to predictive models of emotion [3,4], we can formulate the hypothesis (H1) that participants exposed to reliable contingencies in the learning phase (i.e., the CG) would generalize the learned information to the ambiguous cues of the test phase, thus showing more negative expectancy ratings after the cues that are more perceptually similar to those previously paired with negative pictures. As no extant study directly investigated the interaction between past (un)certainty and cue ambiguity, we tested whether cue predictive meaning modulated expectancies as an exploratory analysis. Moreover, according to previous literature [26] and to what specified in the pre-registration, we expected that ambiguous cues would elicit (H2) less strong generalization effects on expectancy ratings as compared to unambiguous cues and (H3) more unpleasant valence ratings. We also tested (H4) whether cue ambiguity modulates arousal ratings as a function of previous experience.

#### Participants

We used the provider platform Prolific (Prolific, Oxford, UK; www.prolific.co) to recruit 125 adult participants in August 2021. Participants were screened for vision difficulties, including color blindness. Researchers had access only to the following participant’s personal information: Prolific IDs, age, gender, country of birth, country of residence, nationality, employment status and languages spoken. Thus, no identifying information was provided and collected. We estimated the required sample size through an a priori pre-registered (https://osf.io/gdr3b/) simulation-based power analysis for generalized linear mixed-effects models (GLMMs) (R package: simr; [34]). We estimated parameters from data of our previous study (Experiment 1, N = 185 [17]). Pre-registered exclusion criteria were the following: scoring lower than 75% accuracy on attention check items (see below) (N of discarded participants = 0), reporting experienced technical issues in more than 25% of the experimental trials (N = 1). We also excluded data from 3 participants because of data collection failure.

The final sample included 121 participants (58 males, age: M = 25.03, SD = 7.67, range = 18–58; CG N = 57, UG N = 52). All participants gave their written informed consent before starting the experiment, and were paid £1.93 for their participation. All experimental procedures were conducted in accordance with the Declaration of Helsinki, and approved by the local Ethical Committee (protocol no. 4177).

#### Stimulus material and procedure

We employed two emotional S1-S2 paradigms as learning and test phase, respectively (as in [18]). In both phases, unambiguous S1s were red and blue 1-cm-diameter circles. In the test phase only, new ambiguous S1s were introduced unbeknownst to participants: same-sized reddish and bluish (i.e., coral- and turquoise-colored) circles. In both learning and test phases, S2s were colored 800 × 600 px pictures from Nencki Affective Picture System (NAPS) [35], whose valence was manipulated at two levels: negative (Neg; e.g., dead animals, injured people, human threat/war scenes) vs. neutral (Neu; e.g., urban or natural landscapes, people jogging or sitting, animals standing still) (see S1 Table for a list of NAPS pictures employed as S2s). Negative and neutral pictures did not differ in luminance, contrast, complexity and color space indices (see S2 Table), and each picture content (i.e., animals, faces, landscapes, objects, people) was equally represented within each valence level. In attention check trials, a 1-cm yellow circle was used as S1, while a 800 × 600 px picture of a black stripes pattern displayed on a transparent background was used as S2. The expected valence of upcoming S2s (*expectancy ratings*) and subjective affective responses to S2s (*valence* and *arousal ratings*) were assessed through three distinct Visual Analogue Scales (VASs) ranging from 0% to 100%. In the expectancy VAS 0% corresponded to “I definitely expect to see a neutral picture”, 50% represented not knowing what to expect, and 100% corresponded to “I definitely expect to see a negative picture”. In the valence VAS 0% represented “very negative” valence, 50% “neutral” valence, and 100% “very positive” valence. In the arousal VAS 0% meant “relaxed”, 50% represented an intermediate level of activation, and 100% meant “aroused”.

We ran the experiment online, through OpenSesame [36] and the JATOS hosting server [37]. Participants were asked to run the study on a computer, to sit alone in a silent and private room, and to avoid distractions and interruptions, in order to ensure optimal conditions for participation. They were also asked to avoid that someone else could view their screen during the experiment, due to the involvement of emotionally salient material.

Before the learning phase, participants were randomly assigned to the certain group (CG) or the uncertain group (UG). At the beginning of the learning phase, participants received the following instructions: they were asked to look at the screen and pay attention to the relationship between S1 color and S2 valence, and they were asked to press the ‘spacebar’ as fast as they could each time they saw a yellow circle (attention check trials). A practice session of 4 trials followed the instructions: here, participants received feedback on their performance to one attention check trial. After the practice, the learning session started. In each trial S1 was presented first for 250 msec, and it was displayed on a gray background. This was followed by a fixed interstimulus interval (ISI) of 1000 msec, in which the screen remained gray. Then, the S2 was presented for 1000 msec. A white fixation cross was displayed in the center of the screen during the inter-trial interval (ITI), whose length randomly varied between 800 and 1200 msec. The total number of learning trials was 40, presented in random sequence. During this phase, CG participants were exposed to a certain predictive relationship between S1 and S2: each S1 color (i.e., red and blue) was paired with the same S2 valence in 100% of the trials (100% S1-S2 congruency). UG participants, instead, were presented with an uncertain relationship: each S1 color was paired with negative S2s in 50% of the trials, and with neutral S2s in the other 50% (50% S1-S2 congruency). Color-valence pairings were counterbalanced between subjects, and participants were left uninstructed about the S1-S2 predictive ratios.

**Fig 1:**
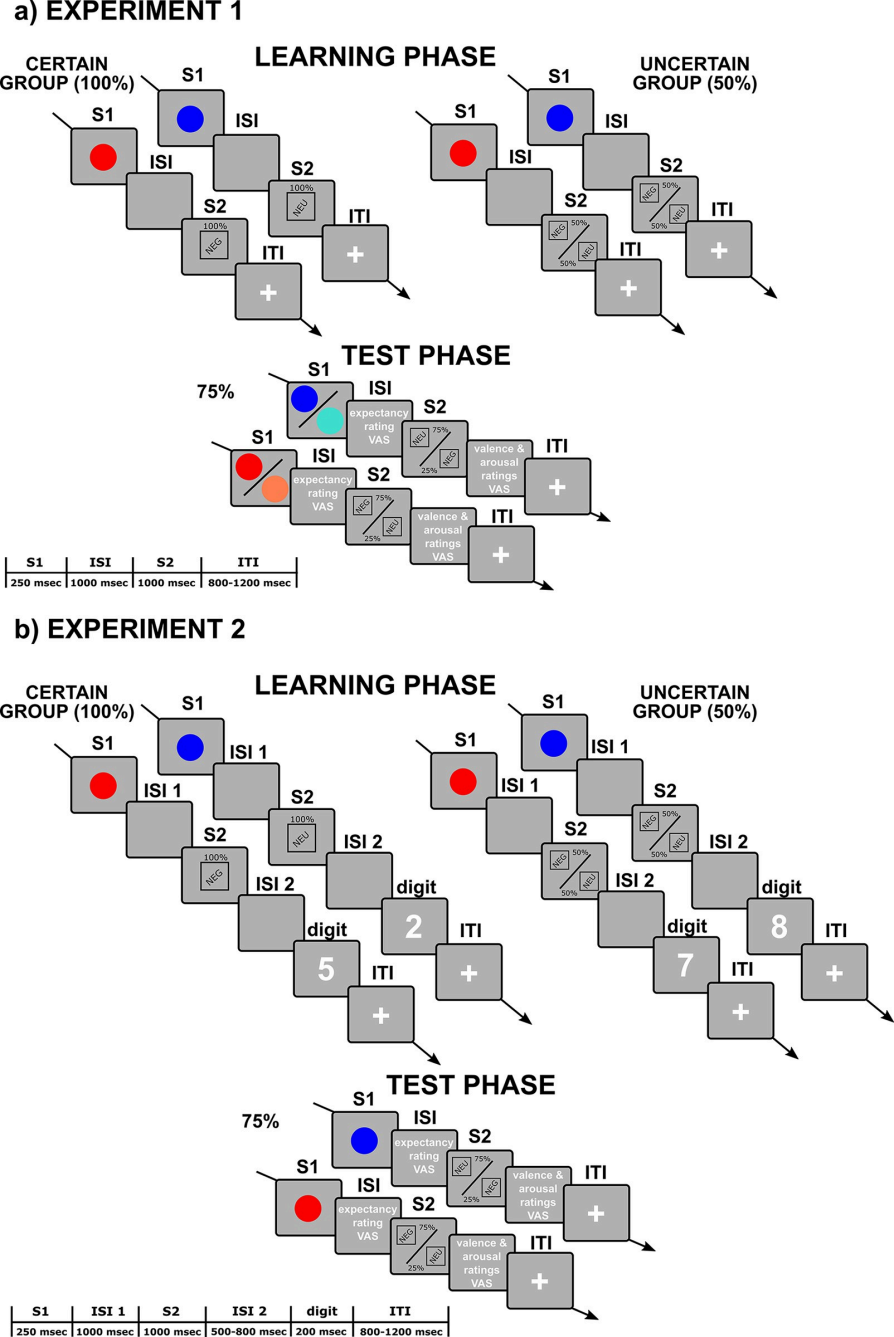
**a)** Example sequence of events and their duration for a trial of Experiment 1, according to the phase (learning, test), and the group (CG, UG). During the learning phase participants experienced a 100% (CG) vs. 50% (UG) affective contingency between S1 color (red or blue) and S2 valence (Neg or Neu), according to group assignment. During the test phase all participants were presented with unambiguous (i.e., red or blue) and ambiguous (i.e., coral or turquoise) S1s, and the S1-S2 affective contingency was fixed at 75%. Participants were asked to answer the VASs either during the ISI for half of the trials (expectancy ratings), or right after the S2 for the other half of the trials (valence and arousal ratings). Response times were self-paced. **b)** Example sequence of events and their duration for a trial of Experiment 2, according to the phase (learning, test), and the group (CG, UG). During the learning phase participants experienced a 100% (CG) vs 50% (UG) affective contingency between S1 color (red or blue) and S2 valence (Neg or Neu), according to group assignment. Then, after the S2, they were presented with the parity judgment task. During the test phase the S1-S2 affective contingency was fixed at 75%. Participants were asked to answer the VASs either during the ISI for half of the trials (expectancy ratings), or right after the S2 for the other half of the trials (valence and arousal ratings). Response times were self-paced. ISI = inter-stimulus interval, ITI = inter-trial interval, VAS = visual analogue scale. The text and the pictures are not drawn to scale.

After the learning phase, a 1-minute interval followed in which participants were asked to wait and relax. Then, both groups were introduced to the same test phase. Instructions of the test phase were to look at the screen and try to predict S2 valence based on S1 color. Participants were told that in some trials they would be asked their expectancy (i.e., to rate how much they expected to see a negative picture after the S1) on a 0–100% scale; while in other trials they would be asked their subjective valence and arousal to the S2, on a 0–100% scale for each dimension. They were asked to give either their expectancy ratings during the ISI, or their valence and arousal ratings right after the S2. VASs response times were self-paced, and the two rating conditions were balanced for the number of trials (50%:50%) and randomly delivered. We presented the expectancy and the valence/arousal ratings in different trials to prevent the two kinds of ratings from influencing each other. Participants were lastly reminded to press the ‘spacebar’ as fast as they could each time they saw the yellow circle (attention check). After the instructions, participants performed a practice session of 4 trials, in which they were trained to give their ratings, and received performance feedback on a single attention check. Then the test session started, with the same trial structure and timing as the learning phase, and a total number of 80 trials. The order of the trials was randomized. *Cue color* was manipulated within participants at four levels: red, blue, coral, and turquoise. Test trials in which we employed the same S1 colors as the learning phase were considered unambiguous (N = 40), whereas test trials in which we employed the new reddish and bluish colors were considered ambiguous (N = 40). *Cue ambiguity*, thus, included two levels: unambiguous (red and blue S1s) vs. ambiguous (coral and turquoise S1s). Participants were not warned about the exposure to new colors. In the test phase, S1 color was moderately predictive of S2 valence, since S1-S2 congruency was fixed at 75% (same S1 color-S2 valence pairings in 75% of the trials). Color-valence pairings were counterbalanced between subjects, and participants were left uninstructed about the S1-S2 predictive ratio. Notably, the new 75% ratio implied that CG participants moved from a reliable predictive relationship (100%, experienced during the learning phase), to a new, more uncertain context (in which the previously learned predictive models were sometimes violated). UG participants, instead, moved from an unreliable predictive relationship (50%), to a more predictive context. Moreover, the 75% is equidistant from the predictive ratios experienced by the CG and the UG (it lies in the middle between 100% and 50%; see [18] for further details on this choice). Fig 1A shows a schematic representation of the experimental paradigm.

After the test phase, participants were redirected to a Qualtrics survey (Qualtrics, Provo, UT; www.qualtrics.com), in which they were asked demographic information (age, gender) and some mood and trait measures. In particular, participants completed the Intolerance of Uncertainty Scale (IUS-12) [38] and the Depression, Anxiety and Stress Scale (DASS-21) [39], as intolerance of uncertainty as well as negative affectivity are known to be potential moderating factors impacting affective predictions [40–44]. Also, participants were asked a forced-choice question about whether and in how many trials they had experienced any technical issues with the Internet connection and/or with pictures uploading (response options: “No, everything worked fine!”, “Yes, in less than 25% of the trials”, “Yes, between 25% and 50% of the trials”, “Yes, between 50% and 75% of the trials”, “Yes, in more than 75% of the trials”). At the end of the survey, participants were thanked, and redirected to Prolific to receive their payment. The experiment lasted about 30 minutes.

### Data analysis

The study had a 2 (*group*, between-subjects: CG vs. UG) × 2 (*cue ambiguity*, within-subjects: ambiguous vs. unambiguous) × 2 (*S2 valence*, within-subjects: Neg vs. Neu) mixed design. The analysis plan was pre-registered on OSF (https://osf.io/gdr3b/).

As pre-registered, univariate outliers (i.e., expectancy ratings) were detected through Median Absolute Deviation values (MAD > 3), and multivariate outliers (i.e., valence and arousal ratings) through the Mahalanobis-Minimum Covariance Determinant (MMCD, breakdown point 0.25) (R package: Routliers; [45]). We identified 12 univariate outliers, and we removed them from data analysis. We also identified 21 multivariate outliers. However, from the visual inspection of their ratings, they emerged as “potentially interesting outliers” (see [45]), since they showed only a slightly different relationship between valence and arousal ratings as compared to other participants. Thus, for this reason and given that none of them significantly impacted the models’ estimates (as assessed through Cook’s distance, see below), we chose to keep their data into the analysis. Overall, data from 109 participants were included in the analyses.

To test our hypotheses (H1, H2, H3, H4), we fitted the following linear mixed-effects models (LMMs) for each dependent variable (DV) (R package: lme4; [46]):

1. expectancy ratings (H1): group, cue color and their interaction as fixed factors, and random slopes for cue color within participants;
2. expectancy ratings (H2): *group*, *cue ambiguity* and their interaction as fixed factors, random slopes for *cue ambiguity* within participant;
3. valence ratings (H3): *group*, *cue ambiguity*, *S2 valence* and their interaction as fixed factors, random slopes for *S2 valence* within participant;
4. arousal ratings (H4): group, cue ambiguity, S2 valence and their interaction as fixed factors, random slopes for S2 valence within participant.

The factor “cue ambiguity” codes for the ambiguity of the cue (within-subjects: ambiguous—that is, coral or turquoise S1 color vs. unambiguous—that is, red or blue S1 color). In the analysis script (shared in OSF https://osf.io/gdr3b/) this factor was called “cue”. Here, for the sake of clarity, we renamed it as “cue ambiguity”. The factor “cue color” codes for the predictive meaning of the cue according to the specific color-valence pairings presented to participants in the test phase (within-subjects: red and coral—that is, a cue that preceded negative stimuli in the test phase vs. blue and turquoise—that is, a cue that preceded neutral stimuli in the test phase). In the analysis script (shared in OSF https://osf.io/gdr3b/) this factor was called “S1 color”. Here, for the sake of clarity, we renamed it as “cue color”. Please note that to compensate for the counterbalancing of S1 color–S2 valence pairings, in the analysis we re-coded as red all the unambiguous cues preceding negative pictures, as coral all the ambiguous cues preceding negative pictures, as blue all the unambiguous cues preceding neutral pictures, and as turquoise all the ambiguous cues preceding neutral pictures, irrespective of the actual color of the cue (see [18] for further details on the cue factor).

The other pre-registered exploratory analyses regarding the effects of time, S2 congruency (expected vs. unexpected S2s), IUS, and DASS-21 questionnaires are reported in S7–S8 Tables. Since the results of these analyses are mainly null, or redundant with respect to both the confirmatory models and the results of our previous work [18], these analyses will not be discussed further in the present manuscript. As pre-registered, for each model we evaluated influential cases through Cook’s distance (>1). No influential cases emerged. As pre-registered, models effects were tested by means of *F*-test and *p*-values, calculated via Satterthwaite’s degrees of freedom method (α = .05, R package: lmerTest; [47]). For each model we reported the estimated parameters with 95% confidence intervals (CI), marginal and conditional R^2^ (estimated as in [48]).

### Results

Descriptive statistics are provided in Table 1.

**Table 1:** Descriptive statistics of Experiment 1.

Group	Cue ambiguity	S2 valence	Expectancy ratings		Valence ratings		Arousal ratings	
			*M*	*SD*	*M*	*SD*	*M*	*SD*
CG	Ambiguous	Neg	52.59	28.74	35.51	29.41	54.98	25.63
		Neu	48.23	28.64	56.31	29.9	42.1	26.49
	Unambiguous	Neg	57.18	30.62	31.62	27.36	58.01	25.07
		Neu	48.95	30.5	56.36	28.47	41.17	26.12
UG	Ambiguous	Neg	52.74	25.38	35.56	27.26	55.08	23.16
		Neu	48.76	26.4	54.07	26.47	44.89	23.64
	Unambiguous	Neg	54.03	26	32.96	25.96	57.99	22.84
		Neu	50.13	25.88	55.73	24.99	43.16	22.74

For each group (CG, UG) and experimental condition (ambiguous/unambiguous cue, negative/neutral S2 valence) we report the mean (M) and standard deviation (SD) of expectancy, valence and arousal ratings.

**Fig 2:**
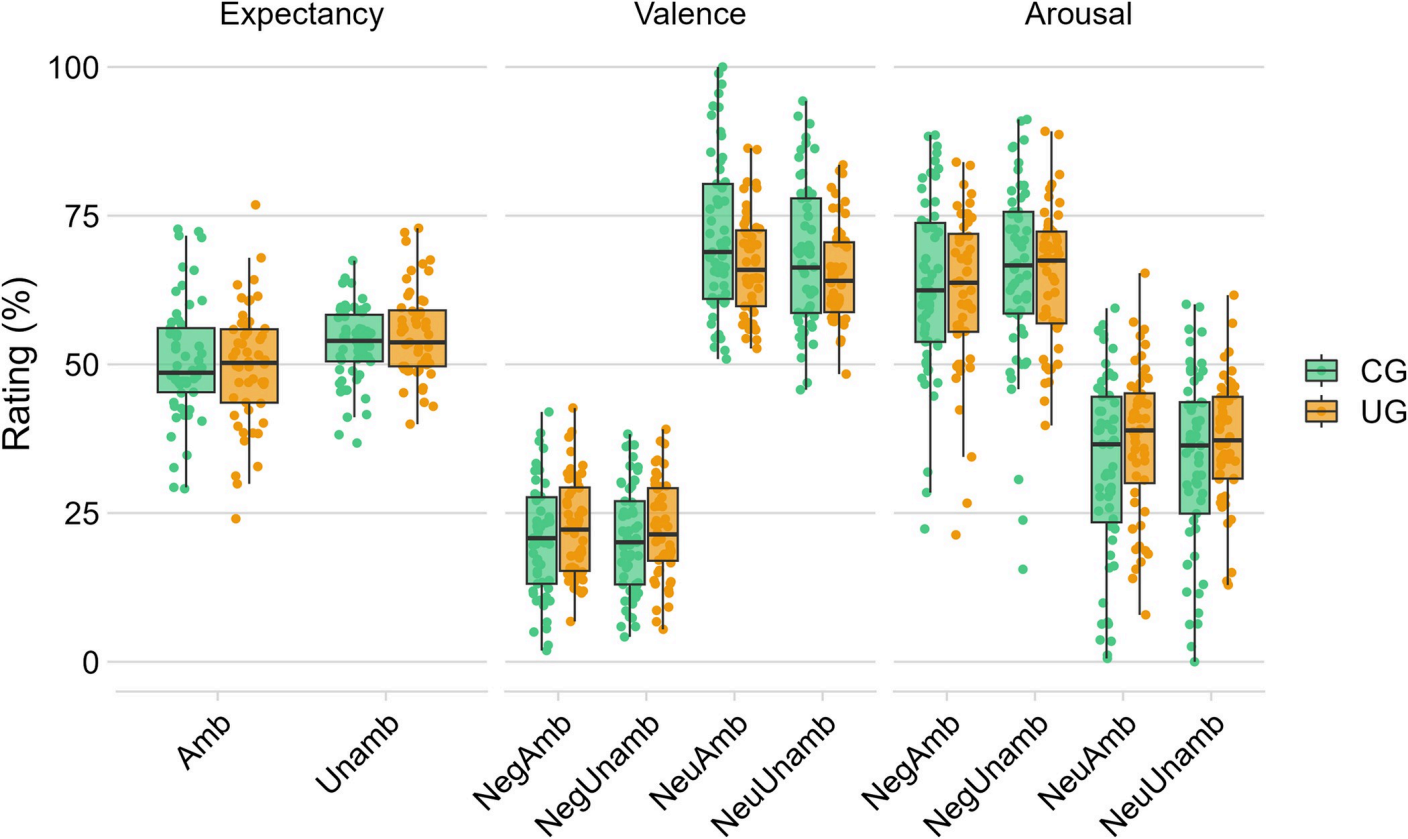
Box-plot of *expectancy*, *valence* and *arousal ratings* in Experiment 1 according to the *group* (CG vs. UG), *cue ambiguity* (Amb-ambiguous vs. Unamb-unambiguous), and *S2 valence* (Neg vs. Neu, for valence and arousal ratings only). Points represent the mean estimated value for each participant and condition.

All the models are summarized in Table 2 and Fig 2. For the expectancy model testing H1 (R^2^ marginal = 0.190, R^2^ conditional = 0.452), we found a main effect of *cue color* (*F*(3, 107) = 33.24, *p* < .001), better specified by a significant interaction between *group* and *cue color* (*F*(3, 107) = 4.05, *p* = .009). Post-hoc contrasts confirmed our previous results [18], showing that participants in the CG reported significantly more extreme expectancy ratings as compared with participants in the UG, but only in the case of unambiguous cues. Indeed, the CG showed more negative expectancy ratings than the UG after red cues (i.e., unambiguous cues preceding negative pictures; CG—UG = 6.78, SE = 3.05, *t*(107) = 2.22, *p* = .028), but not after coral cues (i.e., ambiguous cues preceding negative pictures; CG—UG = 5.19, SE = 2.68, *t*(107) = 1.93, *p* = .056); and it showed less negative expectancy ratings than the UG after blue cues (i.e., unambiguous cues preceding neutral pictures; CG—UG = -10.07, SE = 3.10, *t*(107) = -3.25, *p* = .002), but not after turquoise cues (i.e., ambiguous cues preceding neutral pictures; CG—UG = -3.46, SE = 3.31, *t*(107) = -1.05, *p* = .297). Thus, contrary to H1, we did not find evidence for a strong generalization of the learned predictive meaning of cues to new, ambiguous ones. For the expectancy model testing H2 (R^2^ marginal = 0.006, R^2^ conditional = 0.056), we found a main effect of cue ambiguity (F(1, 107) = 13.19, p < .001): ambiguous cues elicited less negative expectancy ratings than unambiguous ones (ambiguous—unambiguous = -4.08, t(107) = -3.63, p < .001, 95% CI = [-6.31, -1.85]). Thus, the expectancy model supports H2, suggesting a reduced generalization on expectancy ratings to ambiguous cues within both groups.

**Table 2:** Results of LMMs on *expectancy*, *valence* and *arousal ratings* in Experiment 1.

Model	Parameter	Estimate	SE	Statistic	df	p	95% CI	
Expectancy (H1)	Intercept	52.17	0.61	85.91	107.01	< 0.001	50.97	53.38
	UG–CG	0.39	1.21	0.32	107.01	0.746	-2.01	2.80
	Coral–Blue	19.00	2.39	7.96	107.00	< 0.001	14.27	23.72
	Red–Blue	25.91	2.76	9.38	107.21	< 0.001	20.43	31.38
	Turquoise–Blue	-1.25	1.50	-0.83	107.05	0.407	-4.23	1.73
	UG–CG x Coral–Blue	-15.26	4.77	-3.20	107.00	0.002	-24.72	-5.80
	UG–CG x Red–Blue	-16.85	5.53	-3.05	107.21	0.003	-27.81	-5.90
	UG–CG x Turquoise–Blue	-6.61	3.01	-2.20	107.05	0.03	-12.58	-0.65
	σ ID	5.40						
	σ Coral–Blue	23.06						
	σ Red–Blue	27.09						
	σ Turquoise–Blue	12.59						
	σ residual	20.82						
Expectancy (H2)	Intercept	52.17	0.61	85.91	107.00	< 0.001	50.97	53.38
	UG–CG	-0.39	1.21	-0.32	107.00	0.746	-2.80	2.01
	Amb–Unamb	-4.08	1.12	-3.63	107.00	< 0.001	-6.31	-1.85
	group x cue	2.51	2.25	1.12	107.00	0.266	-1.94	6.97
	σ ID	4.62						
	σ cue	7.89						
	σ residual	27.27						
Valence (H3)	Intercept	44.67	0.49	90.57	107.53	< 0.001	43.70	45.65
	UG–CG	0.98	0.99	0.99	107.53	0.322	-0.97	2.94
	Amb–Unamb	1.40	0.49	2.87	4,138.00	0.004	0.45	2.36
	neg–neu	-46.35	1.47	-31.58	107.23	< 0.001	-49.26	-43.44
	group x cue	0.85	0.98	0.87	4,138.00	0.383	-1.06	2.77
	valence x group	-5.50	2.94	-1.87	107.23	0.064	-11.32	0.32
	cue x valence	-2.67	0.98	-2.73	4,138.00	0.006	-4.58	-0.75
	group x cue x valence	-2.40	1.95	-1.23	4,138.00	0.219	-6.23	1.43
	σ ID	4.47						
	σ valence	14.43						
	σ residual	16.03						
Arousal (H4)	Intercept	49.59	0.86	57.89	107.19	< 0.001	47.89	51.29
	UG–CG	-1.62	1.71	-0.95	107.19	0.346	-5.02	1.78
	Amb–Unamb	-1.52	0.51	-2.97	4,138.00	0.003	-2.53	-0.52
	neg–neu	28.71	1.83	15.68	107.17	< 0.001	25.08	32.34
	group x cue	-0.35	1.02	-0.34	4,138.00	0.733	-2.36	1.66
	valence x group	4.67	3.66	1.27	107.17	0.205	-2.59	11.93
	cue x valence	-1.66	1.02	-1.62	4,138.00	0.105	-3.67	0.35
	group x cue x valence	1.69	2.05	0.83	4,138.00	0.409	-2.32	5.70
	σ ID	8.53						
	σ valence	18.34						
	σ residual	16.80						

For each model, we reported the unstandardized regression coefficients, standard errors (SE), 95% confidence intervals (CI), and the associated *t*-test.

For the *valence* model testing H3 (R^2^ marginal = 0.621, R^2^ conditional = 0.704), we found a main effect of *cue ambiguity* (*F*(1, 4138) = 8.26, *p* = .004), and a main effect of *S2 valence* (*F*(1, 107) = 997.35, *p* < .001), better specified by a significant interaction between *cue ambiguity* and *S2 valence* (*F*(1, 4138) = 7.45, *p* = .006). In particular, we found evidence for less unpleasant valence ratings to neutral pictures presented after ambiguous cues as compared to unambiguous ones (Neu: ambiguous vs. unambiguous = 2.74, *t*(4138) = 3.96, *p* < .001, 95% *CI* = [1.38, 4.09]), while valence ratings to negative pictures were not affected by cue ambiguity (Neg: ambiguous–unambiguous = 0.071, *t*(4138) = 0.103, *p* = .918, 95% *CI* = [-1.28, 1.43]). Thus, cue ambiguity actually modulated valence ratings in both groups, but in the opposite direction as H3, with ambiguous cues leading subsequent neutral pictures to elicit less unpleasant subjective ratings.

For the *arousal* model testing H4 (R^2^ marginal = 0.325, R^2^ conditional = 0.566), we found a main effect of *S2 valence* (*F*(1, 107) = 245.75, *p* < .001), with negative pictures eliciting higher arousal ratings than neutral pictures (Neg-Neu = 28.7, SE = 1.83, *t*(107) = 15.68, *p* < .001). We also found a main effect of *cue ambiguity* (*F*(1, 4138) = 8.85, *p* = .003): ambiguous cues elicited lower arousal ratings than unambiguous cues (ambiguous–unambiguous = -1.52, SE = 0.51, *t*(4138) = -2.98, *p* = .003).

### Discussion

The findings obtained in Experiment 1 suggest that no strong generalization of subjective expectancies from the specific features of the learning phase to the new ambiguous features of the test phase emerged. Indeed, when testing the effects of cue predictive meaning (i.e., cue color) on expectancy ratings, we replicated our previous results [18] showing that participants in the CG reported more extreme expectancies than the UG, but only for unambiguous cues. This may seem in contrast with predictive models of emotion [3,4] (see H1), since one may think that an efficient predictive model must be generalizable across different contexts. However, recent contributions [49–51] have highlighted how affective predictions are inherently and indissolubly context-sensitive, being tight to the specific situation in which they develop. Thus, as an alternative explanation consistent with previous literature [26], it may be that the novelty of the stimuli employed as ambiguous cues in the testing phase (namely, current information) prevails over past information in shaping subjective affective ratings. Moreover, *cue ambiguity* per se (regardless of cue predictive meaning) did not interact with uncertainty of previous experience in shaping subjective affective ratings. In fact, no significant group by cue interaction was found in any of the models. We found instead that ambiguous cues elicited less negative expectancy ratings in the *generation*-*implementation* stage, less unpleasant valence to neutral stimuli and overall lower arousal in the *updating* stage, independently from previous learning. Thus, cue ambiguity appears to elicit a dampened subjective affective experience at all stages.

With regards to subjective expectancy (i.e., expectancy ratings) these results are consistent with our hypothesis (see H2, [26]), with regards to subjective reactions to new stimuli (i.e., valence and arousal ratings) they are instead partially in contrast (see H3, [26]). It must be noted, however, that in our paradigm we manipulated previous experience in a separate learning phase and we asked for a trial-by-trial rating of experienced valence (and arousal) to S2s, whereas Chen and Lovibond [26] did not employ a learning paradigm and asked for a post-experiment mood rating. Thus, the different nature of the paradigm and the ratings requested to participants may be accountable for the opposing effects found. Moreover, another S1-S2 study [52] manipulating ambiguity of the target affective pictures, found that ambiguous pictures were rated as less unpleasant and less arousing than unambiguous pictures, coherently with what we found following ambiguous cues. These effects can be explained in light of the time-related distinction between ambiguity and uncertainty (as proposed in [53]). Ambiguity, on the one hand, refers to a static feature of the *here and now*, embedded in the present moment: an ambiguous situation or stimulus is characterized by novelty, unpredictability and also uncertainty [53]. Uncertainty, on the other hand, refers to a *future-oriented* feature, characterized by unpredictability but not necessarily also by ambiguity [53,54]. In Experiment 1, we manipulated uncertainty during the learning phase (where participants were not asked for any rating), and ambiguity during the test phase (where we asked for subjective affective ratings). As a consequence, and in line with the results of our previous research [18], it might be that *current* (i.e., ambiguous vs. unambiguous) information prevailed over *past* information (i.e., certain vs. uncertain) in shaping subjective affective experience. Moreover, it is interesting to note that the role of cue ambiguity in dampening subjective valence was exclusively expressed with regards to neutral stimuli. This may be due to a ceiling effect in valence ratings elicited by negative pictures, that may have covered any modulating contribution of cue ambiguity. Neutral pictures, instead, elicited more variable valence ratings, allowing more subtle modulations of cue ambiguity to statistically emerge.

## Experiment 2

### Method

Experiment 2 investigated whether experiencing *implicit* certain vs. uncertain probabilistic relationships between stimuli might influence subjective ratings to future affective predictions. More specifically, to ensure an implicit exposure to the probabilistic relationship between S1 and S2, during the learning phase we engaged participants in a distracting task (a parity judgment task, see Stimulus material and procedure below for details). Then, we tested if participants were able to implicitly extract the (un)certain probabilistic information available during the learning phase, and to use it in the test phase to rate the expected valence of new affective events, or the subjective valence and arousal to new affective stimuli.

We pre-registered the study on OSF (https://osf.io/z5esb/). We performed confirmatory analyses to test seven pre-registered hypotheses. The first two hypotheses concerned the behavioral performance to the parity judgment task. We hypothesized to find (H1a) faster *RTs* in the CG as compared to the UG, and (H1b) no differences in *accuracy* between the groups [22]. This would be in line with the predictive models’ assumption that the two contingencies (100% and 50%) can be experienced implicitly without any significant difference between them [6]. The remaining hypotheses regarded the test phase. As a third hypothesis (H2a), we expected to find more negative *expectancy ratings* in the UG as compared to the CG [21,22,24,55,56]. The fourth hypothesis (H2b), opposed to H2a, was that participants in the CG would show more negative expectancy ratings after the cues which were previously paired with a negative picture during the learning phase [18]. This hypothesis is coherent with predictive models of emotion [3,4], according to which we can expect that contingencies of the learning phase would be implicitly inferred. The fifth hypothesis (H3a) was that participants in the UG would show higher *arousal* and more unpleasant *valence ratings* to S2s than participants in the CG [33]. The sixth hypothesis (H3b), opposed to H3a, was that participants in the CG would show higher arousal and more unpleasant valence ratings to S2s, as compared to participants in the UG [27,29–32]. The last hypothesis (H3c), opposed to both H3a and H3b, was to find only a main effect of S2 valence, with significantly higher arousal and more unpleasant valence ratings to negative S2s independently from the experimental group [18,23].

### Participants

We computed the required sample size through an a priori pre-registered (https://osf.io/z5esb/) power analysis for GLMMs (see 2.1.1), estimating parameters from pilot data (N = 18). We recruited 125 adult participants through the provider platform Prolific (Prolific, Oxford, UK; www.prolific.co) in August 2021. Participants were screened for vision difficulties, including color blindness. Researchers had access only to the following participant’s personal information: Prolific IDs, age, gender, country of birth, country of residence, nationality, employment status and languages spoken. Thus, no identifying information was provided and collected. In order to be included in Experiment 2, participants should not have participated in Experiment 1. Data from 9 participants were discarded according to the pre-registered exclusion criteria: scoring lower than -2 SD from the mean accuracy in the parity judgment task (N = 7), reporting internet/uploading issues in more than 25% of the experimental trials (N = 2), reporting to have caught the exact probabilistic relationship between S1 color and S2 valence during the learning phase (N = 0). The final sample included 116 participants (58 males, age: M = 25.06, SD = 7.63, range = 18–55; CG N = 55, UG N = 54). All participants gave their written informed consent before starting the experiment, and were paid £2.13 for their participation. All experimental procedures were conducted in accordance with the Declaration of Helsinki, and approved by the local Ethical Committee (protocol no. 4177).

### Stimulus material and procedure

Materials and procedures were the same as in Experiment 1 (see Stimulus Material and Procedure above), but in Experiment 2 we employed only unambiguous (i.e., red and blue) cues. During the learning phase a parity judgment task was introduced. Single digits from 1 to 9 were employed as stimuli. Participants were informed that they would see a sequence of stimuli on the screen: a colored circle, followed by a picture, and then a number. They were instructed to look at the screen and judge if the number was odd or even, by pressing the ‘Z’ or ‘M’ keys. Response keys were counterbalanced between subjects. Instructions were followed by a practice session of 3 trials, in which participants were trained to give their parity judgments, and they received feedback on their performance. Then, the learning session started, with the same trials’ structure, timing and number as in Experiment 1. In each trial, after the S2, a second ISI with a random duration between 500 and 800 msec followed, in which the screen remained gray. Then, a random digit between 1 and 9 was presented in the center of the screen for 200 msec, and participants had up to 1500 msec to judge the digit’s parity by pressing the ‘Z’ or ‘M’ keys. Participants were left uninstructed about the S1-S2 ratios (100% for the CG, 50% for the UG). Fig 1B shows a schematic representation of the experimental paradigm.

At the end of the test phase, participants were directed to the Qualtrics survey (see Stimulus Material and Procedure above). Here, we added an open question as a manipulation check: participants were asked whether they caught any relationship between the color of the circle and the affective valence of the pictures during the learning phase. Participants were then redirected back to Prolific to receive their payment. The experiment lasted about 30 minutes.

### Data analysis

The study had a 2 (*group*, between-subjects: CG vs. UG) × 2 (*S2 valence*, within-subjects: Neg vs. Neu) mixed design. The analysis plan was pre-registered on OSF (https://osf.io/z5esb/).

During data pre-processing, participants’ verbatim responses to the manipulation check question (see Stimulus Material and Procedure above) were qualitatively analyzed by the experimenter, and coded as “yes” or “no” according to their content. No response was coded as “yes”, suggesting that no participants reported to have caught the probabilistic relationship between S1 and S2.

Following the pre-registered procedures for outliers detection and management, we detected 5 univariate outliers (MAD > 3) that were excluded from data analysis. We also detected 36 multivariate outliers (MMCD, breakdown point 0.25). From the visual inspection of their ratings it emerged that 2 of them had reversed the scales’ poles (“error outliers”, see [45]), and they were therefore removed from data analysis. The remaining multivariate outliers showed only a slightly different relationship between valence and arousal ratings as compared to other participants, thus we chose to keep them into data analysis (“potentially interesting outliers”, see [45]), since none of them impacted the models’ estimates (as assessed through Cook’s distance). Overall, data from 109 participants were included in the analyses.

Before performing the analyses, we pre-processed RTs to the parity judgment task according to the following pre-registered steps: (i) trimming RTs between 100 and 1500 msec [57]; (ii) discarding RTs of incorrect trials; (iii) adjusting RTs for the speed-accuracy trade off by means of Inverse Efficiency Score (IES) transformation [58]; (iv) log-transforming IES to account for their skewed distribution [57,59].

In order to test our a-priori hypotheses (H1a and H1b on parity judgment task; H2a, H2b, H3a, H3b and H3c on test phase ratings), for each DV we fitted the following (G)LMMs (R package: lme4; [46]):

1. log-transformed IES (H1a): *group* as fixed factor, random intercept for participant;
2. accuracy (H1b): logistic regression with *group* as fixed factor, random intercept for participant;
3. expectancy ratings (H2a, H2b): *group*, *cue* (within-subjects: cue_neg_ vs. cue_neu_) and their interaction as fixed factors, random slopes for *cue* within participant;
4. valence and arousal ratings (H3a, H3b, H3c): *group*, *S2 valence* and their interaction as fixed factors, random slopes for S2 valence within participant.

The factor “cue” codes for the predictive meaning of the cue according to the specific color-valence pairings experienced by participants in the test phase (within-subjects: cue_neg_−that is, a cue that preceded negative stimuli in the test phase vs. cue_neu_−that is, a cue that preceded neutral stimuli in the test phase). In the analysis script (shared on OSF https://osf.io/z5esb/) this factor was called “S1 color”. Here, for the sake of clarity, we renamed it as “cue” (see [18] and above for further details on the cue factor, and on how we compensated for the counterbalancing of S1 color–S2 valence pairings in the analysis).

The pre-registered exploratory analyses regarding the effects of time, S2 congruency (expected vs. unexpected S2s), IUS, and DASS-21 questionnaires are reported in S3–S6 Tables. Since the results of these analyses are mainly null, or redundant with respect to both the confirmatory models reported below and the results of our previous work [18], these analyses will not be discussed further in the present manuscript.

Influential cases for each model, as well as the methods to test model’s effects were pre-registered. For each model no influential cases emerged, as evaluated through Cook’s distance (>1). LMMs effects were tested by means of *F*-test and *p*-values, calculated via Satterthwaite’s degrees of freedom method (α = .05, R package: lmerTest; [47]), GLMMs effects were evaluated through Type II Analysis of Deviance (R package: car; [60]). For each model we reported the estimated parameters with 95% CI, marginal and conditional R^2^ (estimated as in [48]).

### Results

Descriptive statistics are provided in Table 3.

**Table 3:** Descriptive statistics of Experiment 2.

Group	Cue	S2 valence	Expectancy ratings		Valence ratings		Arousal ratings	
			*M*	*SD*	*M*	*SD*	*M*	*SD*
CG	cue_neg_	Neg	56.78	25.98	32.56	28.44	56.64	25.02
		Neu	57.93	25.2	58.51	28.48	39.35	25.95
	cue_neu_	Neg	48.65	26.38	36.21	28.24	55.4	24.91
		Neu	49.34	25.73	56.28	28.23	41.3	24.76
UG	cue_neg_	Neg	57.1	29	33.23	28.84	56.16	25.89
		Neu	58.08	28.8	59.38	29.49	39.89	25.55
	cue_neu_	Neg	48.44	30.67	35.87	28.22	54.2	25.53
		Neu	49.8	29.35	58.3	28.4	40.65	25.02

For each group (CG, UG) and experimental condition (cue_neg_/cue_neu_, negative/neutral S2 valence) we report the mean (M) and standard deviation (SD) of expectancy, valence and arousal ratings.

**Fig 3:**
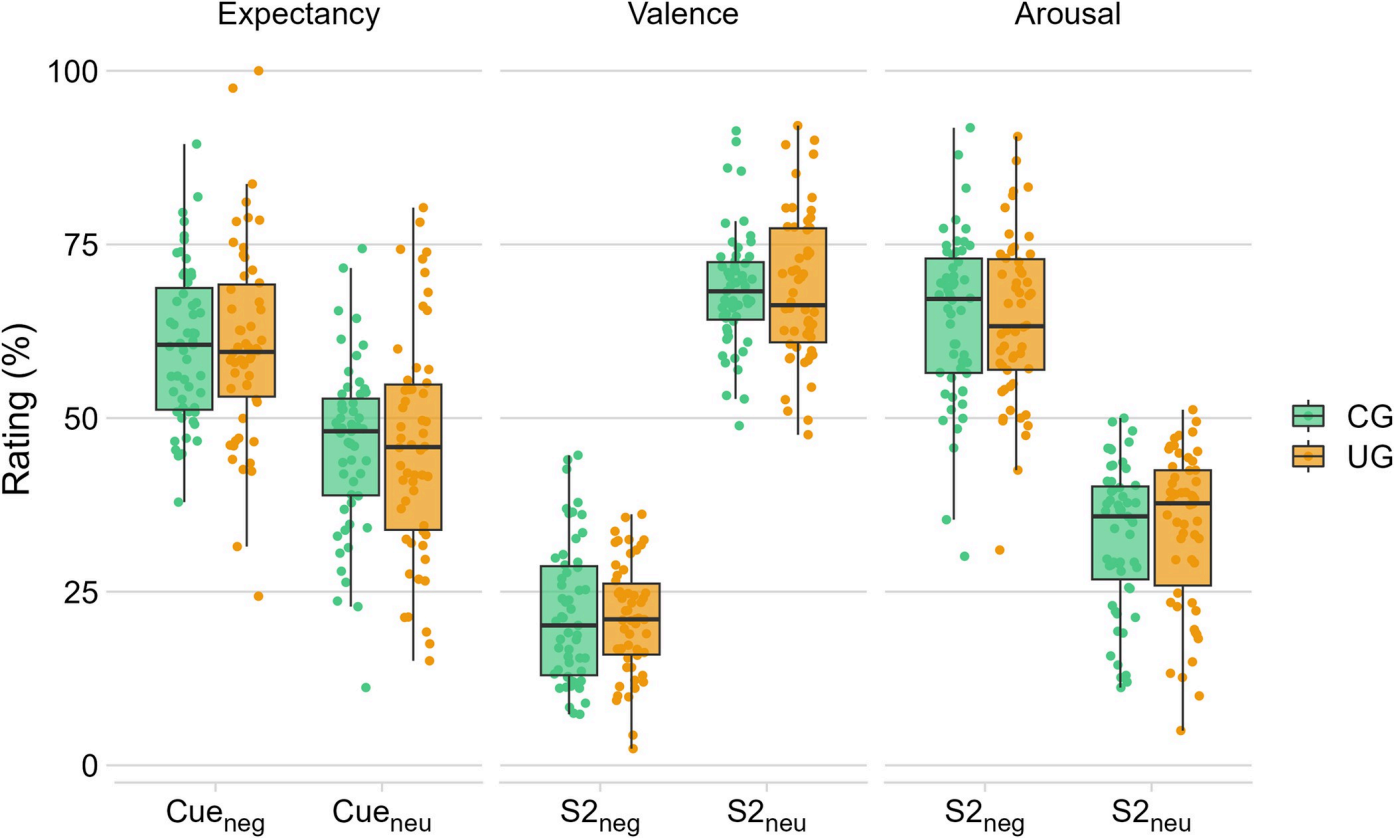
Box-plot of *expectancy*, *valence* and *arousal ratings* in Experiment 2 according to the *group* (CG vs. UG), and the *cue* (Cue_Neg_ vs. Cue_Neu_, for expectancy ratings) or the *S2 valence* (Neg vs. Neu, for valence and arousal ratings). Points represent the mean estimated value for each participant and condition.

All the models are summarized in Table 4 and Fig 3. For the *IES* model testing H1a (R^2^ marginal = 0.003, R^2^ conditional = 0.968), we did not find any effect of *group* (*F*(1, 105) = 0.33, *p* = .565). Thus, the IES model does not support the hypothesis of faster RTs in the CG as compared to the UG (H1a). We found the same result for the *accuracy* model testing H1b (R^2^ marginal = 0.009, R^2^ conditional = 0.486), with no significant effect of *group* (χ^2^ = 1.71, *p* = .191). Therefore, we confirmed our hypothesis (H1b) of not finding group differences in accuracy scores to the parity judgment task.

**Table 4:** Results of confirmatory (G)LMMs on *IES*, *accuracy*, *expectancy*, *valence* and *arousal ratings* in Experiment 2.

Model	Parameter	Estimate	SE	Statistic	df	p	95% CI	
IES (H1a)	Intercept	6.61	0.08	84.06	105.24	< 0.001	6.46	6.77
	UG—CG	-0.09	0.16	-0.58	105.24	0.565	-0.40	0.22
	σ ID	0.82						
	σ residual	0.15						
Accuracy (H1b)	Intercept	2.35	0.18	12.95		< 0.001	2.00	2.71
	UG—CG	0.47	0.36	1.31		0.191	-0.23	1.18
	σ ID	1.75						
Expectancy (H2a, H2b)	Intercept	53.36	0.85	62.75	107.01	< 0.001	51.68	55.05
	UG—CG	-0.17	1.70	-0.10	107.01	0.922	-3.54	3.20
	cue_neg_—cue_neu_	14.55	1.96	7.42	107.00	< 0.001	10.66	18.44
	cue x group	-0.24	3.92	-0.06	107.00	0.952	-8.02	7.54
	σ ID	8.03						
	σ cue	19.04						
	σ residual	23.92						
Valence (H3a, H3b, H3c)	Intercept	44.99	0.55	82.53	107.02	< 0.001	43.91	46.07
	UG—CG	0.24	1.09	0.22	107.02	0.83	-1.93	2.40
	Neg—Neu	-47.10	1.42	-33.19	107.02	< 0.001	-49.91	-44.28
	valence x group	0.79	2.84	0.28	107.02	0.782	-4.84	6.41
	σ ID	4.98						
	σ valence	13.75						
	σ residual	17.45						
Arousal (H3a, H3b, H3c)	Intercept	48.95	0.76	64.77	106.96	< 0.001	47.45	50.45
	UG—CG	-0.49	1.51	-0.33	106.96	0.745	-3.49	2.50
	Neg—Neu	30.86	1.57	19.60	107.01	< 0.001	27.74	33.98
	valence x group	1.47	3.15	0.47	107.01	0.641	-4.77	7.72
	σ ID	7.36						
	σ valence	15.42						
	σ residual	18.03						

For each model we reported the estimates (unstandardized regression coefficients for LMMs, odds ratio for GLMMs), SE, 95% CI, and the associated statistics (*t*-test for LMMs, χ2 for GLMMs).

For the *expectancy* model testing H2a vs. H2b (R^2^ marginal = 0.068, R^2^ conditional = 0.267), we only found a main effect of *cue* (*F*(1, 107) = 54.98, *p* < .001): in both groups, participants showed significantly more negative expectancy ratings after the cue_neg_ (i.e., cues preceding negative pictures in the test phase) than after the cue_neu_ (i.e., cues preceding neutral pictures in the test phase) (cue_neg_ vs. cue_neu_ = 14.5, SE = 1.96, *t*(107) = 7.42, *p* < .001). Thus, the expectancy model does not support either of our two hypotheses (H2a, H2b).

For both *valence* (R^2^ marginal = 0.596, R^2^ conditional = 0.673) and *arousal* (R^2^ marginal = 0.352, R^2^ conditional = 0.52) models testing H3a vs. H3b vs. H3c, we found a main effect of *S2 valence*, with both groups reporting significantly greater unpleasantness (*F*(1, 107) = 1101.78, *p* < .001; Neg vs. Neu = -47.1, SE = 1.42, *t*(107) = -33.19, *p* < .001) and higher arousal (*F*(1, 107) = 384.03, *p* < .001; Neg vs. Neu = 30.9, SE = 1.57, *t*(107) = 19.6, *p* < .001) towards negative pictures. Thus, valence and arousal models support the hypothesis of no group differences in S2-ratings (H3c).

### Discussion

Experiment 2 replicated our previous findings [18] for valence and arousal, but not for expectancy ratings. Indeed, both groups showed similar expectancies, predicting upcoming pictures’ valence according to the 75% contingencies of the test phase rather than to previously (and implicitly) experienced contingencies. Thus, the results did not support the hypothesis that probabilistic information can be implicitly extracted and subsequently used to modulate subjective affective ratings, as it can be expected according to predictive models of emotion [3,4,6]. They are however consistent with alternative explanations derived from some recent evidence [61], according to which awareness seems to be a necessary precursor for learning, especially in the case of repetition learning.

Remarkably, we can reasonably exclude any potential difference in the way the two groups experienced the implicit contingencies during the learning phase. In fact, no significant group differences emerged on the parity judgment task, as measured by RTs (see H1a) and accuracy (see H1b), suggesting that the distracting task acted similarly in the two groups. Demonstrating this is crucial, as judgments among low levels of contingency (e.g., 50%) are more difficult than judgments among high levels of contingency (e.g., 100%) [62], and this could have (but did not) exert confounding effects on how contingency learning occurred in the two groups.

## General discussion

The studies reported in the present paper attempted to shed further light on the construction of subjective affective experience as conceived within the predictive framework [3,4]. Indeed, little is known about how subjective experience is shaped by new and potentially ambiguous environmental cues, or whether it can be influenced by implicit previous learnings, despite both being considered crucial factors for constructing efficient predictions [1,2].

Regarding cue ambiguity (Experiment 1), we found that ambiguous cues elicited a dampened subjective affective experience in all the prediction stages, independently from previous learnings. Interestingly, when presented with unambiguous cues, participants formerly exposed to certain contingencies (i.e., the CG, that constructed a reliable prediction) showed more extreme expectancy ratings than participants exposed to uncertainty (i.e., the UG). This fully replicates our previous results [18], and may be due to a pre-activation of the expected affective experience, as suggested by predictive models of emotion [3,4]. When faced with ambiguous cues, instead, participants were unable to implement a reliable prediction and coherently pre-activate the associated subjective experience, and thus the latter was dampened.

Concerning previous implicit experience (Experiment 2), we did not find any evidence that subjective affective ratings are shaped by (un)certainty of previous implicit learning. Indeed, all participants reported similar subjective experience. This is consistent with some recent evidence about repetition learning, providing strong evidence that awareness is a necessary precursor for learning [61], and suggests that affective stimuli require a prompt response primarily driven by a quick bottom-up evaluation of present inputs rather than by top-down predictions based on past information. However, this does not necessarily exclude that an implicit extraction of statistical regularities from task stimuli contingencies occurred in some ways (e.g., at a covert level, measured as patterns of neural/psychophysiological activity). There is a bunch of evidence, indeed, suggesting that implicit learning may potentially occur across all the statistical learning paradigms, and that these two learning processes are associated with the activation of partially overlapping neural networks [63,64]. In spite of this, our result reasonably suggests that, without any explicit focus of attention on previous experience, the effect of the latter does not emerge at the subjective level significantly shaping the reported affective experience. Future investigations should clarify whether implicit learning can shape affective prediction construction at other levels.

Overall, our results advance the understanding of the mechanisms underlying subjective affective experience as constructed from prior knowledge. Crucially, we fully replicated our previous results [18], collecting additional evidence that subjective expectancies are sensitive to *uninstructed* (but not implicit) statistical regularities experienced in the past. With this, we further supported predictive models of emotion [3,4]: certain past learning experiences are used to construct highly reliable predictions, thus leading to a coherent pre-activation of the associated subjective experience. However, our results suggest some caution when applying predictive processing theoretical principles to the affective domain, especially with regards to the updating stage. Indeed, we did not find evidence for a generalization effect to new and potentially ambiguous stimuli, nor evidence of implicitness. Predictive processing theories, instead, assume that prediction updating should be modulated by stimuli predictability [11,13,20,65]. Nonetheless, in recent studies it was consistently found that affective processing during the updating stage is not (or is only weakly) influenced by stimulus predictability both at the subjective [18] and at the neural levels [20,41]. Thus, when it comes to updating, more weight seems to be attributed to the affective nature of stimuli themselves and to the contextual information available in the *current* moment, rather than to top-down predictions based on *past* experiences. This may be due to the evolutionary relevance of affective stimuli, in that a quick bottom-up evaluation of present inputs may cover a more efficient role in promoting survival (and thus, it must be prioritized).

As a selected broader implication of our study, consistent with predictive models of emotion [3,4], we argue that a paradigm shift is needed in the study of emotions, in the sense that we further supported the idea that emotions cannot be studied independently from the specific context in which they are constructed [3,49,66]. We found, indeed, that previous experience failed to generalize to new contexts in an experimental setting which is rather artificial as compared to everyday life and which implies the use of symbolic and simple stimuli. This highlights the importance of taking context into careful account when studying affective processes in complex situations and/or beyond each specific experimental setup.

Notwithstanding, some limitations of our research are worth mentioning. First, we did not assess individual differences in cognitive processes that may influence affective predictions (e.g., attention, memory, etc.), so we cannot exclude that these processes may have exerted influencing effects. However, we can exclude that mood and personality traits modulating affective predictions (i.e., intolerance of uncertainty and negative affectivity, that we measured and controlled for in our analyses; see S5–S6, S9 and S10 Tables) exerted confounding effects. Also, there are some constraints on the generality of our findings [67] since our sample only included WEIRD participants. The lack of participants from other cultural groups precludes our ability to ascertain whether our findings have a broader generalizability. Second, our paradigm remains quite artificial with respect to real-life situations, in which people often experience multimodal and dynamic affective stimuli. Third, previous experience is manipulated only in terms of (un)certain probabilistic relationships between stimuli (i.e., S1-S2 congruency). However, other characteristics of previous experience, such as the frequency of exposure, or the familiarity with the physical environment in which the stimuli are embedded, may be of interest, too. Fourth, we have only manipulated ambiguity with respect to cues, but also ambiguity of targets can have a great impact on subjective affective experience. Last, in our study we only focused on subjective experience as overtly rated by participants (since we were not able to collect covert -e.g., psychophysiological- measures due to COVID-19 pandemic restrictions), but more subtle modulations may be experimentally captured by measuring also covert processing indices. We thus encourage future studies to complement our results, by integrating both subjective and objective measures of affective processing.

## Supporting information

S1 Table List of NAPS picture names used as S2s in Experiment 1 and 2, sorted by valence (Neg = negative, Neu = neutral). (PDF)

S2 Table Means (M), standard deviations (SD), and results of two-tailed t-tests assuming unequal variance in luminance, contrast, complexity indices (i.e., JPEG size, entropy), and color space indices (i.e., LABL, LABA, LABB), referred to negative (Neg) and neutral (Neu) NAPS pictures employed as S2s in Experiment 1 and 2. (PDF)

S3 Table Pre-registered exploratory models on Block effect in Experiment 1. (PDF)

S4 Table Pre-registered exploratory models on S2 Congruency effect in Experiment 1. (PDF)

S5 Table Pre-registered exploratory models on Intolerance of Uncertainty Scale (IUS) effect in Experiment 1. (PDF)

S6 Table Pre-registered exploratory models on Depression, Anxiety and Stress Scale (DASS-21) effect in Experiment 1. (PDF)

S7 Table Pre-registered exploratory models on Block effect in Experiment 2. (PDF)

S8 Table Pre-registered exploratory models on S2 Congruency effect in Experiment 2. (PDF)

S9 Table Pre-registered exploratory models on Intolerance of Uncertainty Scale (IUS) effect in Experiment 2. (PDF)

S10 Table Pre-registered exploratory models on Depression, Anxiety and Stress Scale (DASS-21) effect in Experiment 2. (PDF)
