# Supplementary material for: How previous experience shapes future affective subjective ratings: A follow-up study investigating implicit learning and cue ambiguity
Source: PLoS One. 2024 Feb 9;19(2):e0297954. doi: 10.1371/journal.pone.0297954 (PMC10857730; doi:10.1371/journal.pone.0297954)
Supplement: S1 Table — (PDF) [file pone.0297954.s001.pdf]

Supporting Information

How previous experience shapes future affective subjective ratings: a follow-up study investigating implicit learning and cue ambiguity

Valence	NAPS picture names
Neg	Animals_001_h; Animals_024_h; Animals_025_h; Animals_027_h; Animals_033_h; Animals_038_h; Animals_054_h; Animals_068_h; Animals_071_h; Animals_074_h; Animals_077_h; Animals_078_h; Faces_146_h; Faces_150_h; Faces_152_h; Faces_170_h; Faces_271_h; Faces_272_h; Faces_285_h; Faces_290_h; Faces_291_h; Faces_293_h; Faces_294_h; Faces_302_h; Landscapes_002_h; Landscapes_004_h; Landscapes_005_h; Landscapes_007_h; Landscapes_010_h; Landscapes_011_h; Landscapes_014_h; Landscapes_017_h; Landscapes_022_h; Landscapes_026_h; Landscapes_139_h; Landscapes_177_h; Objects_001_h; Objects_002_h; Objects_003_h; Objects_007_h; Objects_011_h; Objects_022_h; Objects_125_h; Objects_132_h; Objects_139_h; Objects_149_h; Objects_283_h; Objects_285_h; People_001_h; People_008_h; People_020_h; People_022_h; People_118_h; People_127_h; People_136_h; People_140_h; People_200_h; People_215_h; People_225_h; People_226_h
Neu	Animals_109_h; Animals_114_h; Animals_122_h; Animals_125_h; Animals_126_h; Animals_136_h; Animals_165_h; Animals_169_h; Animals_170_h; Animals_197_h; Animals_202_h; Animals_206_h; Faces_184_h; Faces_186_h; Faces_188_h; Faces_282_h; Faces_304_h; Faces_314_h; Faces_316_h; Faces_326_h; Faces_329_h; Faces_331_h; Faces_335_h; Faces_343_h; Landscapes_009_h; Landscapes_041_h; Landscapes_048_h; Landscapes_050_h; Landscapes_089_h; Landscapes_100_h; Landscapes_107_h; Landscapes_127_h; Landscapes_143_h; Landscapes_149_h; Landscapes_163_h; Landscapes_172_h; Objects_025_h; Objects_033_h; Objects_041_h; Objects_069_h; Objects_075_h; Objects_078_h; Objects_079_h; Objects_103_h; Objects_254_h; Objects_262_h; Objects_263_h; Objects_270_h; People_069_h; People_089_h; People_099_h; People_101_h; People_109_h; People_153_h; People_162_h; People_167_h; People_173_h; People_178_h; People_194_h; People_250_h

S1 Table. List of NAPS picture names used as S2s in Experiment 1 and 2, sorted by valence (Neg = negative, Neu = neutral).
