# Supplementary material for: How previous experience shapes future affective subjective ratings: A follow-up study investigating implicit learning and cue ambiguity
Source: PLoS One. 2024 Feb 9;19(2):e0297954. doi: 10.1371/journal.pone.0297954 (PMC10857730; doi:10.1371/journal.pone.0297954)
Supplement: S2 Table — (PDF) [file pone.0297954.s002.pdf]

Supporting Information

How previous experience shapes future affective subjective ratings: a follow-up study investigating implicit learning and cue ambiguity

Measure	Neg		Neu		t(118)	*p*
	M	*SD*	*M*	*SD*		
luminance	114.149	27.503	116.943	27.615	-0.555	.58
contrast	65.682	11.569	66.225	11.490	-0.258	.797
jpeg_size	350012.367	118541.318	331739.683	120579.766	0.837	.404
entropy	7.579	0.338	7.608	0.311	-0.489	.626
LABL	47.078	11.035	48.411	11.090	-0.66	.511
LABA	1.684	4.6	0.541	7.901	0.969	.335
LABB	7.444	9.766	7.492	11.416	-0.025	.980

S2 Table. Means (*M*), standard deviations (*SD*), and results of two-tailed *t*-tests assuming unequal variance in luminance, contrast, complexity indices (i.e., JPEG size, entropy), and color space indices (i.e., LABL, LABA, LABB), referred to negative (Neg) and neutral (Neu) NAPS pictures employed as S2s in Experiment 1 and 2.
