# Supplementary material for: How previous experience shapes future affective subjective ratings: A follow-up study investigating implicit learning and cue ambiguity
Source: PLoS One. 2024 Feb 9;19(2):e0297954. doi: 10.1371/journal.pone.0297954 (PMC10857730; doi:10.1371/journal.pone.0297954)
Supplement: S3 Table — (PDF) [file pone.0297954.s003.pdf]

Supporting Information

How previous experience shapes future affective subjective ratings: a follow-up study investigating implicit learning and cue ambiguity

	Expectancy ratings			Valence ratings			Arousal ratings		
Predictors	*Estimate*	*CI*	*p*	*Estimate*	*CI*	*p*	*Estimate*	*CI*	*p*
Group	-0.41	-2.78 – 1.96	0.736	1.01	-0.93 – 2.95	0.307	-1.64	-4.99 – 1.72	0.340
Block	-1.65	-3.29 – -0.01	**0.048**	-0.12	-1.09 – 0.85	0.805	-0.64	-1.67 – 0.38	0.217
Cue ambiguity	-4.08	-6.28 – -1.89	**<0.001**	1.45	0.50 – 2.40	**0.003**	-1.52	-2.53 – -0.52	**0.003**
S2 Valence				-46.37	-49.25 – -43.48	**<0.001**	28.74	25.14 – 32.33	**<0.001**
Group x Block	-1.05	-4.33 – 2.23	0.532	0.32	-1.62 – 2.27	0.743	2.51	0.47 – 4.55	**0.016**
Group x Cue ambiguity	2.50	-1.89 – 6.89	0.264	0.86	-1.05 – 2.77	0.376	-0.40	-2.41 – 1.60	0.694
Block x Cue ambiguity	-1.71	-4.99 – 1.57	0.308	1.10	-0.84 – 3.05	0.267	-1.43	-3.48 – 0.62	0.172
Group x S2 Valence				-5.42	-11.19 – 0.36	0.066	4.57	-2.62 – 11.75	0.213
Block x S2 Valence				-5.65	-7.59 – -3.71	**<0.001**	3.27	1.23 – 5.32	**0.002**
Cue ambiguity x S2 Valence				-2.58	-4.49 – -0.67	**0.008**	-1.66	-3.67 – 0.34	0.104
Group x Block x Cue ambiguity	-3.03	-9.60 – 3.53	0.365	-2.01	-5.90 – 1.89	0.312	1.40	-2.70 – 5.50	0.504
Group x Block x S2 Valence				-2.01	-5.89 – 1.87	0.309	1.26	-2.83 – 5.34	0.546
Group x Cue ambiguity x S2 Valence				-2.43	-6.25 – 1.38	0.211	1.61	-2.40 – 5.62	0.431
Block x Cue ambiguity x S2 Valence				2.77	-1.12 – 6.67	0.162	-2.38	-6.49 – 1.72	0.254

Group x Block x Cue ambiguity x S2 Valence		-1.51	-9.30 – 6.27	0.703	-0.20	-8.40 – 8.00	0.961
Marginal R^2 / Conditional R^2	0.007 / 0.057	0.624 / 0.707			0.327 / 0.568		

S3 Table. Pre-registered exploratory models on Block effect in Experiment 1.

For the *expectancy* model, we found a main effect of Block (F(1, 4278) = 3.91, p = .048): participants showed more negative expectancy ratings in the second block as compared to the first block (block 1 vs. block 2 = -1.65, SE = 0.84, t(4278) = -1.98, p = .048).

For the *valence* model, we found a Block x S2 Valence interaction (F(1, 4192) = 32.66, p < .001): negative stimuli were rated as less unpleasant in the second block as compared to the first block (block 1 vs. block 2 = -2.95, SE = 0.7, t(4191) = -4.22, p < .001), whereas neutral stimuli were rated as less pleasant in the second block as compared to the first block (block 1 vs. block 2 = 2.70, SE = 0.7, t(4175) = 3.86, p < .001).

For the *arousal* model, we found a Group x Block interaction (F(1, 4157) = 5.79, p = .016): participants in the UG only showed heightened arousal ratings in the second block as compared to the first block (CG – block 1 vs. block 2 = 0.61, SE = 0.72, t(4161) = 0.85, p = .398; UG – block 1 vs. block 2 = -1.90, SE = 0.75, t(4153) = -2.53, p = .012). We also found a Block x S2 Valence interaction (F(1, 4158) = 9.87, p = .002): in the case of neutral stimuli only, participants showed heightened arousal ratings in the second block as compared to the first block (NEG – block 1 vs. block 2 = 0.99, SE = 0.74, t(4156) = 1.35, p = .178; NEU – block 1 vs. block 2 = -2.28, SE = 0.74, t(4157) = -3.1, p = .002).
