# Supplementary material for: How previous experience shapes future affective subjective ratings: A follow-up study investigating implicit learning and cue ambiguity
Source: PLoS One. 2024 Feb 9;19(2):e0297954. doi: 10.1371/journal.pone.0297954 (PMC10857730; doi:10.1371/journal.pone.0297954)
Supplement: S4 Table — (PDF) [file pone.0297954.s004.pdf]

Supporting Information

How previous experience shapes future affective subjective ratings: a follow-up study investigating implicit learning and cue ambiguity

Predictors	Valence ratings			Arousal ratings		
	Estimate	*CI*	*p*	*Estimate*	*CI*	*p*
Group	0.88	-1.14 – 2.89	0.393	-1.57	-4.98 – 1.84	0.367
Cue ambiguity	0.63	-0.47 – 1.74	0.260	-0.36	-1.53 – 0.81	0.546
S2 Valence	-44.91	-47.84 – -41.98	**<0.001**	27.91	24.27 – 31.55	**<0.001**
S2 Congruency	-3.74	-4.85 – -2.64	**<0.001**	1.48	0.31 – 2.65	**0.013**
Group x Cue ambiguity	1.00	-1.21 – 3.21	0.376	-1.15	-3.48 – 1.19	0.336
Group x S2 Valence	-5.18	-11.04 – 0.69	0.084	4.30	-2.98 – 11.58	0.247
Cue ambiguity x S2 Valence	-3.34	-5.56 – -1.13	**0.003**	-1.99	-4.32 – 0.35	0.096
Group x S2 Congruency	0.44	-1.77 – 2.65	0.697	-0.36	-2.69 – 1.98	0.764
Cue ambiguity x S2 Congruency	3.79	1.58 – 6.01	**0.001**	-4.90	-7.24 – -2.57	**<0.001**
S2 Valence x S2 Congruency	-5.90	-8.11 – -3.69	**<0.001**	3.13	0.79 – 5.46	**0.009**
Group x Cue ambiguity x S2 Valence	-0.61	-5.04 – 3.81	0.786	1.39	-3.29 – 6.06	0.561
Group x Cue ambiguity x S2 Congruency	-0.67	-5.09 – 3.76	0.768	3.23	-1.44 – 7.90	0.175
Group x S2 Valence x S2 Congruency	-0.93	-5.35 – 3.49	0.680	1.43	-3.24 – 6.10	0.550
Cue ambiguity x S2 Valence x S2 Congruency	3.87	-0.56 – 8.29	0.087	0.66	-4.01 – 5.33	0.782
Group x Cue ambiguity x S2 Valence x S2 Congruency	-6.90	-15.74 – 1.95	0.126	0.92	-8.42 – 10.26	0.847
Marginal R^2 / Conditional R^2	0.628 / 0.711			0.328 / 0.569		

S4 Table. Pre-registered exploratory models on S2 Congruency effect in Experiment 1.

For the *valence* model, we found a main effect of S2 Congruency ($F(1, 4130) = 44.06$, $p < .001$), suggesting that congruent stimuli elicited more unpleasant valence ratings than incongruent stimuli (Cong vs. Incong = -3.74, SE = 0.56, $t(4130) = -6.64$, $p < .001$). This effect was better specified by significant Cue ambiguity x S2 Congruency ($F(1, 4130) = 11.32$, $p < .001$) and S2 valence x S2 Congruency ($F(1, 4130) = 27.38$, $p < .001$) interactions. Post-hoc comparisons revealed that congruent stimuli elicited more unpleasant valence

ratings than incongruent stimuli both in the case of ambiguous (Cong vs. Incong = -1.85, SE = 0.86, $t(4130) = -2.14$, $p = .032$) and unambiguous cues (Cong vs. Incong = -5.64, SE = 0.73, $t(4130) = -7.75$, $p < .001$). Moreover, congruent negative stimuli elicited more unpleasant valence ratings than incongruent negative stimuli (Cong vs. Incong = -6.69, SE = 0.8, $t(4130) = -8.39$, $p < .001$), whereas no significant difference emerged between congruent and incongruent stimuli in the case of neutral S2s (Cong vs. Incong = -0.79, SE = 0.8, $t(4130) = -0.99$, $p = .32$).

For the *arousal* model, also, we found a main effect of S2 Congruency ($F(1, 4130) = 6.16$, $p = .013$), suggesting that congruent stimuli elicited higher arousal ratings than incongruent stimuli (Cong vs. Incong = 1.48, SE = 0.6, $t(4130) = 2.48$, $p = .013$). This effect was better specified by significant Cue ambiguity x S2 Congruency ($F(1, 4130) = 16.94$, $p < .001$) and S2 Valence x S2 Congruency ($F(1, 4130) = 6.88$, $p = .009$) interactions. Post-hoc contrasts showed that congruent stimuli elicited higher arousal ratings than incongruent stimuli only when following unambiguous cues (Ambiguous cues – Cong vs. Incong = -0.97, SE = 0.91, $t(4130) = -1.07$, $p = .285$; Unambiguous cues – Cong vs. Incong = 3.93, SE = 0.77, $t(4130) = 5.11$, $p < .001$). Furthermore, congruent negative stimuli elicited higher arousal ratings than incongruent negative stimuli (Cong vs. Incong = 3.04, SE = 0.84, $t(4130) = 3.61$, $p < .001$), whereas no significant difference emerged between congruent and incongruent stimuli in the case of neutral S2s (Cong vs. Incong = -0.08, SE = 0.84, $t(4130) = -0.1$, $p = .92$).
