# Supplementary material for: How previous experience shapes future affective subjective ratings: A follow-up study investigating implicit learning and cue ambiguity
Source: PLoS One. 2024 Feb 9;19(2):e0297954. doi: 10.1371/journal.pone.0297954 (PMC10857730; doi:10.1371/journal.pone.0297954)
Supplement: S5 Table — (PDF) [file pone.0297954.s005.pdf]

Supporting Information

How previous experience shapes future affective subjective ratings: a follow-up study investigating implicit learning and cue ambiguity

Predictors	Expectancy ratings			Valence ratings			Arousal ratings		
	Estimate	*CI*	*p*	*Estimate*	*CI*	*p*	*Estimate*	*CI*	*p*
Group	-0.70	-3.19 – 1.78	0.580	0.98	-1.00 – 2.96	0.330	-1.85	-5.26 – 1.56	0.288
IUS total score	0.12	-0.02 – 0.25	0.090	0.01	-0.10 – 0.11	0.924	0.13	-0.05 – 0.32	0.159
Group x IUS total score	-0.09	-0.36 – 0.17	0.487	-0.08	-0.29 – 0.14	0.487	0.18	-0.19 – 0.55	0.338
Marginal R^2 / Conditional R^2	0.002 / 0.031			0.000 / 0.007			0.004 / 0.105		

S5 Table. Pre-registered exploratory models on Intolerance of Uncertainty Scale (IUS) effect in Experiment 1.

No significant effects emerged.
