# Supplementary material for: How previous experience shapes future affective subjective ratings: A follow-up study investigating implicit learning and cue ambiguity
Source: PLoS One. 2024 Feb 9;19(2):e0297954. doi: 10.1371/journal.pone.0297954 (PMC10857730; doi:10.1371/journal.pone.0297954)
Supplement: S6 Table — (PDF) [file pone.0297954.s006.pdf]

Supporting Information

How previous experience shapes future affective subjective ratings: a follow-up study investigating implicit learning and cue ambiguity

Predictors	Expectancy ratings			Valence ratings			Arousal ratings		
	Estimate	*CI*	*p*	*Estimate*	*CI*	*p*	*Estimate*	*CI*	*p*
Group	-0.93	-3.41 – 1.56	0.464	1.42	-0.46 – 3.29	0.138	-2.18	-5.40 – 1.04	0.184
DASS stress scale	0.09	-0.40 – 0.59	0.711	-0.25	-0.63 – 0.13	0.196	1.07	0.42 – 1.72	**0.001**
DASS depression scale	0.04	-0.31 – 0.39	0.829	0.19	-0.08 – 0.45	0.167	-0.51	-0.97 – -0.06	**0.028**
DASS anxiety scale	0.18	-0.23 – 0.58	0.399	-0.06	-0.37 – 0.25	0.719	-0.22	-0.75 – 0.31	0.416
Group x DASS stress scale	0.14	-0.86 – 1.14	0.784	-0.75	-1.50 – 0.00	0.050	1.60	0.30 – 2.89	**0.015**
Group x DASS depression scale	0.18	-0.52 – 0.88	0.615	0.10	-0.43 – 0.63	0.717	-0.44	-1.35 – 0.47	0.340
Group x DASS anxiety scale	-0.50	-1.31 – 0.32	0.234	0.30	-0.32 – 0.91	0.346	-0.65	-1.71 – 0.41	0.226
Marginal R^2 / Conditional R^2	0.003 / 0.032			0.003 / 0.006			0.022 / 0.106		

S6 Table. Pre-registered exploratory models on Depression, Anxiety and Stress Scale (DASS-21) effect in Experiment 1.

For the *arousal* model, we found main effects of DASS stress ($F(1, 99) = 10.53$, $p = .002$) and depression ($F(1, 99) = 4.85$, $p = .03$) scales: higher stress scores predicted higher arousal ratings, whereas higher depression scores predicted lower arousal ratings. Moreover, we found a significant Group x DASS stress scale interaction ($F(1, 99) = 5.87$, $p = .017$): higher stress scores predicted higher arousal ratings in the CG only ($\beta = 1.87$, $\beta_{SE} = 0.46$, CI = [0.96 – 2.78]), whereas no significant relationship emerged in the UG ($\beta = 0.27$, $\beta_{SE} = 0.47$, CI = [-0.67 – 1.21]).
