# Supplementary material for: How previous experience shapes future affective subjective ratings: A follow-up study investigating implicit learning and cue ambiguity
Source: PLoS One. 2024 Feb 9;19(2):e0297954. doi: 10.1371/journal.pone.0297954 (PMC10857730; doi:10.1371/journal.pone.0297954)
Supplement: S7 Table — (PDF) [file pone.0297954.s007.pdf]

Supporting Information

How previous experience shapes future affective subjective ratings: a follow-up study investigating implicit learning and cue ambiguity

Predictors	**Expectancy ratings**			**Valence ratings**			**Arousal ratings**		
	Estimate	*CI*	*p*	*Estimate*	*CI*	*p*	*Estimate*	*CI*	*p*
Group	-0.16	-3.49 – 3.18	0.926	0.24	-1.90 – 2.37	0.828	-0.48	-3.43 – 2.47	0.748
Block	-0.95	-2.39 – 0.50	0.198	0.53	-0.52 – 1.58	0.325	-2.28	-3.36 – -1.19	**<0.001**
Cue	14.59	10.74 – 18.44	**<0.001**						
S2 Valence				-47.11	-49.89 – -44.33	**<0.001**	-47.11	-49.89 – -44.33	**<0.001**
Group x Block	-0.15	-3.04 – 2.74	0.920	0.51	-1.60 – 2.61	0.638	0.51	-1.60 – 2.61	0.638
Group x Cue	-0.27	-7.98 – 7.43	0.944						
Block x Cue	-3.24	-6.13 – -0.35	**0.028**						
Group x S2 Valence				0.78	-4.78 – 6.35	0.783	1.51	-4.65 – 7.67	0.630
Block x S2 Valence				-0.83	-2.94 – 1.27	0.438	0.50	-1.67 – 2.68	0.650
Group x Block x Cue	4.02	-1.76 – 9.80	0.173						
Group x Block x S2 Valence				-0.16	-4.37 – 4.05	0.941	5.21	0.86 – 9.56	**0.019**
Marginal R^2 / Conditional R^2	0.069 / 0.268			0.596 / 0.673			0.355 / 0.522		

S7 Table. Pre-registered exploratory models on Block effect in Experiment 2.

For the *expectancy* model, we found a significant Block x Cue interaction ($F(1, 4193) = 4.84$, $p = .028$): participants showed more negative expectancy ratings in the second block as compared to the first block only after cues preceding negative pictures (cue_{neg} – block 1 vs. block 2 = -2.57, SE = 1.04, $t(4193) = -2.47$, $p = .014$; cue_{neu} – block 1 vs. block 2 = 0.67, SE = 1.04, $t(4181) = 0.65$, $p = .519$).

For the *valence* model, we did not find any significant effect.

For the *arousal* model, we found a main effect of Block ($F(1, 4176) = 16.86$, $p < .001$), better specified by a significant Group x Block x S2 Valence interaction ($F(1, 4177) = 5.52$, $p = .019$). Participants showed higher arousal ratings in the second block as compared to the first block (block 1 vs. block 2 = -2.28, SE = 0.56, $t(4176) = -4.11$, $p < .001$). Post-hoc contrasts also showed that this difference was significant only in the case of negative stimuli in the UG (block 1 vs. block 2 = -3.74, SE = 1.11, $t(4171) = -3.36$, $p < .001$) and neutral stimuli in the CG (block 1 vs. block 2 = -3.42, SE = 1.11, $t(4183) = -3.09$, $p = .002$), whereas it was not significant in the case of negative stimuli in the CG (block 1 vs. block 2 = -0.31, SE = 1.1, $t(4168) = -0.29$, $p = .776$) and neutral stimuli in the UG (block 1 vs. block 2 = -1.64, SE = 1.11, $t(4177) = -1.47$, $p = .142$).
