# Supplementary material for: How previous experience shapes future affective subjective ratings: A follow-up study investigating implicit learning and cue ambiguity
Source: PLoS One. 2024 Feb 9;19(2):e0297954. doi: 10.1371/journal.pone.0297954 (PMC10857730; doi:10.1371/journal.pone.0297954)
Supplement: S8 Table — (PDF) [file pone.0297954.s008.pdf]

Supporting Information

How previous experience shapes future affective subjective ratings: a follow-up study investigating implicit learning and cue ambiguity

Predictors	Valence ratings			Arousal ratings		
	Estimate	*CI*	*p*	*Estimate*	*CI*	*p*
Group	-0.07	-2.28 – 2.15	0.953	-0.30	-3.32 – 2.73	0.848
S2 Valence	-45.69	-48.53 – -42.84	**<0.001**	30.32	27.17 – 33.47	**<0.001**
S2 Congruency	-4.05	-5.24 – -2.87	**<0.001**	2.38	1.15 – 3.62	**<0.001**
Group x S2 Valence	0.17	-5.52 – 5.86	0.953	1.96	-4.34 – 8.26	0.541
Group x S2 Congruency	1.20	-1.17 – 3.58	0.320	-0.79	-3.26 – 1.68	0.531
S2 Valence x S2 Congruency	-5.64	-8.01 – -3.26	**<0.001**	2.17	-0.30 – 4.64	0.084
Group x S2 Valence x S2 Congruency	2.45	-2.30 – 7.20	0.311	-1.95	-6.89 – 2.98	0.438
Marginal R^2 / Conditional R^2	0.600 / 0.678			0.354 / 0.522		

S8 Table. Pre-registered exploratory models on S2 Congruency effect in Experiment 2.

For the *valence* model, we found a main effect of S2 Congruency ($F(1, 4137) = 44.8$, $p < .001$), better specified by a significant S2 Valence x S2 Congruency interaction ($F(1, 4137) = 21.67$, $p < .001$). Post-hoc tests revealed that congruent stimuli elicited more unpleasant valence ratings than incongruent stimuli (Cong vs. Incong = -4.05, SE = 0.61, $t(4137) = -6.69$, $p < .001$). This difference remains significant only for negative S2s (NEG – Cong vs. Incong = -6.87, SE = 0.86, $t(4137) = -8.02$, $p < .001$), whereas no difference emerged between congruent and incongruent stimuli for neutral S2s (NEU – Cong vs. Incong = -1.23, SE = 0.86, $t(4137) = -1.44$, $p = .15$).

For the arousal model, we only find a main effect of S2 Congruency ($F(1, 4137) = 14.33$, $p < .001$), suggesting that congruent stimuli elicited higher arousal ratings than incongruent stimuli (Cong vs. Incong = 2.38, SE = 0.63, $t(4137) = 3.79$, $p < .001$).
