# Supplementary material for: How previous experience shapes future affective subjective ratings: A follow-up study investigating implicit learning and cue ambiguity
Source: PLoS One. 2024 Feb 9;19(2):e0297954. doi: 10.1371/journal.pone.0297954 (PMC10857730; doi:10.1371/journal.pone.0297954)
Supplement: S9 Table — (PDF) [file pone.0297954.s009.pdf]

Supporting Information

How previous experience shapes future affective subjective ratings: a follow-up study investigating implicit learning and cue ambiguity

Predictors	**Expectancy ratings**			**Valence ratings**			**Arousal ratings**		
	Estimate	*CI*	*p*	*Estimate*	*CI*	*p*	*Estimate*	*CI*	*p*
Group	-0.00	-3.57 – 3.56	0.998	0.14	-2.15 – 2.44	0.904	-0.47	-3.62 – 2.68	0.770
IUS total score	0.03	-0.22 – 0.28	0.814	0.06	-0.10 – 0.23	0.432	0.06	-0.16 – 0.28	0.586
Group x IUS total score	-0.03	-0.53 – 0.48	0.915	0.08	-0.24 – 0.40	0.632	-0.16	-0.60 – 0.29	0.495
Marginal R^2 / Conditional R^2	0.000 / 0.084			0.000 / 0.013			0.001 / 0.074		

S9 Table. Pre-registered exploratory models on Intolerance of Uncertainty Scale (IUS) effect in Experiment 2.

No significant effect emerged.
