# Supplementary material for: How previous experience shapes future affective subjective ratings: A follow-up study investigating implicit learning and cue ambiguity
Source: PLoS One. 2024 Feb 9;19(2):e0297954. doi: 10.1371/journal.pone.0297954 (PMC10857730; doi:10.1371/journal.pone.0297954)
Supplement: S10 Table — (PDF) [file pone.0297954.s010.pdf]

Supporting Information

How previous experience shapes future affective subjective ratings: a follow-up study investigating implicit learning and cue ambiguity

Predictors	Expectancy ratings			Valence ratings			Arousal ratings		
	Estimate	*CI*	*p*	*Estimate*	*CI*	*p*	*Estimate*	*CI*	*p*
Group	0.09	-3.47 – 3.65	0.962	-0.03	-2.30 – 2.23	0.977	0.30	-2.81 – 3.42	0.849
DASS stress scale	0.26	-0.46 – 0.99	0.482	0.19	-0.27 – 0.65	0.427	-0.02	-0.65 – 0.62	0.955
DASS depression scale	-0.14	-0.63 – 0.36	0.590	0.14	-0.18 – 0.46	0.388	-0.39	-0.83 – 0.04	0.076
DASS anxiety scale	0.22	-0.49 – 0.93	0.544	0.07	-0.39 – 0.52	0.776	0.04	-0.58 – 0.66	0.903
Group x DASS stress scale	-1.12	-2.57 – 0.33	0.132	-0.61	-1.53 – 0.31	0.197	-0.32	-1.59 – 0.95	0.621
Group x DASS depression scale	0.94	-0.06 – 1.94	0.064	0.44	-0.20 – 1.07	0.176	-0.57	-1.44 – 0.30	0.201
Group x DASS anxiety scale	0.18	-1.25 – 1.60	0.808	0.08	-0.83 – 0.98	0.868	0.19	-1.06 – 1.43	0.770
Marginal R^2 / Conditional R^2	0.005 / 0.087			0.003 / 0.014			0.008 / 0.078		

S10 Table. Pre-registered exploratory models on Depression, Anxiety and Stress Scale (DASS-21) effect in Experiment 2.

No significant effect emerged.
